# Study Protocol: A retrospective observational analysis of patients treated for hospital-acquired pneumonia.

**DOI:** 10.3310/nihropenres.13853.2

**Published:** 2025-09-15

**Authors:** Samuel Quarton, Mohammed Baragilly, Elizabeth Sapey

**Affiliations:** 1 NIHR Birmingham Biomedical Research Centre, Birmingham, England, UK; 2 Department of Health Informatics, University Hospitals Birmingham NHS Foundation Trust, PIONEER Health Data Research Hub in Acute Care, Birmingham, B15 2WB, UK

**Keywords:** Hospital-acquired pneumonia, antimicrobial stewardship, diagnosis.

## Abstract

**Background:**

Hospital-acquired pneumonia (HAP) is an important complication of hospital admission, with both high incidence and consequences for patients. However, our understanding of causative organisms and prognostic factors is limited. Although ventilator-associated pneumonia (VAP,) an important subset of HAP,has been extensively investigated, less is known about non-ventilated cases, leading to calls for focused research in this group. This retrospective observational cohort study aims to define a population of patients treated as HAP by comparing ventilated and non-ventilated cases. It aims to clarify how often a microbiological diagnosis is reached, what organisms are frequently identified, and whether this has a relevant impact on the outcomes. The relative impact of positive radiographic changes among patients treated for HAP will also be assessed.

**Methods:**

Data will be obtained from the Health Data Research UK acute care hub, ‘PIONEER’ Cases meeting coding criteria or a clinical surveillance definition of HAP over a 5-year period will be extracted. Demographic, clinical, and microbiological variables will be analysed initially descriptively, and subsequently, with multiple logistic regression analysis to investigate factors affecting microbiological diagnosis. Key outcome variables are in-hospital, 30-day and 1 year mortality, as well as all-cause readmissions within 1 year. Secondary outcomes include nosocomial infections, such as *C. difficile*. Kaplan-Meier curves and a Cox proportional hazards regression model will be used to investigate outcomes and compare subgroups. A key comparison is between those in whom a putative pathogen is identified and those treated entirely empirically. For this purpose, we will also compare outcomes using an inverse probability of treatment weighting analysis. Additionally, we will explore identifying consolidation on chest imaging reports using natural language processing to allow consideration of the relative impact this may have on mortality and readmission rates.

## Plain Language Summary

Pneumonia – a chest infection where areas of the lung get filled with pus and inflammation – can develop among people admitted to hospital for other reasons. When this occurs, we call it ‘hospital-acquired pneumonia’ or HAP. This includes people who get pneumonia whilst on a ventilator (or artificial breathing machine), as well as those who are not, despite potentially important differences between these two groups. There is limited data related to pneumonia in non-ventilated patients, meaning there is uncertainty about which viruses and bacteria commonly cause it, and what factors might affect recovery.

Currently, we often don’t find the bacteria or virus causing HAP. This means we can’t give targeted antibiotics, instead having to rely on our ‘best-guess’ treatment, which may not work, or may cause side-effects. We also know that many people are treated as ‘HAP’ despite not having the changes on chest x-ray they would technically need to meet a definition of pneumonia. However it is not clear whether this matters in terms of outcomes, as chest x-rays can be unreliable.

This study therefore hopes to:

Look at any differences between ventilated and non-ventilated patients with pneumonia

Work out how often the bacteria or virus causing infection is found, and whether this affects how often patients recover.

Compare patients who have changes on the chest xray with those that don’t, to see if there are any important differences between these groups.

To investigate these questions, we will access a database of all patients treated at four hospitals over a 5-year period. We will look at any patients labelled as developing a HAP during their hospital stay. This is known to have limited accuracy, so we will also get patients where their data suggests they had a HAP, even if they were not given a formal label.

## Introduction

As the most common nosocomial infection ^1^, hospital-acquired pneumonia (HAP) causes significant harm to individuals and the wider healthcare system. Compared to community-acquired pneumonia, the causative organisms vary more with a greater incidence of antimicrobial resistance ^2, 3^, and so treatment usually involves the use of broad-spectrum antibiotics, with implications for antimicrobial stewardship. Although the significance of HAP is widely recognised, it is poorly defined. Diagnosis is clinical, with varying criteria applied, and no gold-standard test available ^4^. Causative organisms are only identified in a minority of cases, and while case definitions typically require radiological changes in keeping with a pneumonic process ^5^, these have been found to be absent in up to half of patients treated with HAP ^6^, possibly because of the high variability in the interpretation of chest radiographs or the insensitivity of the test ^7, 8^. Furthermore, HAP encompasses both ventilator-associated pneumonia (VAP) and pneumonia occurring among non-ventilated hospitalised patients (NV-HAP). Despite these groups having different demographics and risk factors, research has generally not distinguished between these two subgroups or focused solely on VAP. The NV-HAP group, which accounts for the majority of HAP cases, has been relatively understudied, leading to a lack of clarity regarding clinical, microbiological, and prognostic factors ^9^.

One difference between NV-HAP and VAP is the relative difficulty in obtaining a microbiological diagnosis among nonventilated patients. Unlike VAP, where bronchoalveolar lavage can be performed more readily, obtaining microbiological specimens in NV-HAP often relies on spontaneous expectoration of sputum. This may not be possible because many of the factors predisposing patients to the development of HAP can also make expectoration more difficult. However, the importance of this remains uncertain. In the absence of an organism to target, treatment consists of empirical antibiotics, which intuitively have several drawbacks compared with the use of organism-guided antibiotics. These include uncertainty that patients’ infections are covered by the antibiotic administered, increased harm from side effects associated with broad-spectrum antibiotics, and increased risk of driving antimicrobial resistance. However, evidence for the benefit of obtaining a microbiological diagnosis in this cohort of patients is currently lacking.

Furthermore, as already highlighted, cases treated as HAP are often found on retrospective review, not to have the radiological changes needed to meet the current case definitions. However, the importance of this finding remains unclear. It may be that radiological changes have little to add to clinical decision making if patients are otherwise showing signs of a developing chest infection, such as fever, new productive cough, and raised inflammatory markers. This becomes more plausible considering the reduced negative predictive value of chest radiography when performed in bed-bound patients ^10^, as is frequently the case for HAP. Conversely, the presence or absence of new infiltrates on radiographs may indeed usefully separate the two distinct cohorts of patients with different prognoses. Even if that is the case, one could argue that if patients are being treated pragmatically for HAP regardless of X-ray changes, we should study them on that basis – data related to a case definition that is not routinely applied in clinical practice would have limited relevance.

Therefore, this retrospective cohort study proposes to consider the following broad questions:

1. How do clinical and microbiological characteristics differ between VAP and NV-HAP?
2. How frequently are microbiological causes identified, and is this associated with patient outcomes?
3. Is there a meaningful difference in clinical features or outcomes between those treated as HAP with or without corresponding changes on radiographs?

### Aims

To address the questions identified above, we will interrogate a large retrospective database of patients treated for HAP (both NV-HAP and VAP), investigating the following specific aims:

- Characterise the demographic and clinical characteristics of this patient group.
- Characterise the microbiology of HAP, to include the rates of microbiological sampling, the organisms identified, and whether these were covered by antimicrobials given.
- Compare changes in the above between patients with NV-HAP and VAP and over time.
- Identify factors associated with the likelihood of sending samples for microbiological analysis and with organisms being positively identified.
- Identify factors influencing prognosis in HAP, including comparing outcomes in patients treated with organism-guided antibiotics with those in whom empirical antibiotics alone were used.
- Where it can be established through natural language processing of radiological reports, the difference in outcomes among patients with and without consolidation should be assessed.

The methods and analysis plan used to address these aims are outlined below, followed by a discussion of the potential limitations of this methodology.

## Protocol

### Data sources and variables

Data is provided from PIONEER, the Health Data Research UK Acute Care Data Hub. PIONEER holds acute healthcare data for patients accessing acute care services from four separate hospitals within the University Hospital Birmingham NHS Trust.

Patients aged ≥ 18 or over who were treated at any of the UHB sites in a 5-year period between January 2018 and December 2022 were included. This timeframe allows for outcome data to be reviewed for 1-year post-discharge. The following definitions were used to select the patients:

- Inpatient care episodes were associated with the ICD10 code of J12-18 followed by Y95 or where there was a prior admission in the previous 10 days.
- SNOMED code of 425464007

It has previously been demonstrated that coding data for hospital-acquired pneumonia are often inaccurate. Furthermore, because hospital-acquired pneumonia can occur at any point in the hospital stay after the first 48 hours, identifying the date and time of diagnosis from coding data alone is not possible, limiting the extent of the analysis that can be performed.

Therefore, a further cohort of patients was also included, approximating a clinical definition of hospital-acquired pneumonia using readily available and routinely collected electronic data. This has been adapted from a previously published surveillance definition ^11^ as follows:

1. New antibiotic prescription ^*^ lasting for at least three days (prescribed >48 h after admission)
AND
2. Worsening oxygenation within 24hours of this prescription date (defined as a new oxygen requirement, increase in oxygen flow rate, or increase in FIO2 recorded in observations)
AND
3. Chest imaging was requested within 24 hours of the antibiotic prescription date. (Plain chest radiography, CT thorax, high-resolution CT thorax, or CT pulmonary angiography).

All analysis will be conducted on the combined overall cohort, however sensitivity analysis will also compare patients in each definition.

Patients under the age of 18 will be excluded; otherwise, there are no specific exclusion criteria. To avoid issues related to the non-independence of the results, where patients meet the inclusion criteria on multiple occasions, the first spell alone will be used.

As the period in question covers the Covid-19 pandemic, patients testing positive for covid-19 will be flagged to allow for a sensitivity analysis to be performed, but will not be excluded.

### Patient and Public Involvement

As part of the planning for the Birmingham BRC acute care theme, public workshops were held to discuss which infections patients wanted us to focus on, and hospital-acquired infections, including HAP, were identified as a major priority for patients. Patients also identified that they wanted us to perform research on the management of acute infections in hospitals, including creating better tools to identify those at risk of deterioration during infections.

Once the specific project was developed in detail, it was presented to members of a support group for patients living with long-term lung conditions who discussed the data that might be needed and provided feedback. Several patients in this meeting had experienced HAP, and those that had not, recognized that they were at a higher risk of HAP if they were admitted to the hospital *,* so their perspective on what to prioritise within this project was extremely relevant. They emphasised the importance of looking for factors that could be changed or lead to improved outcomes. The resulting data request form was then reviewed by the PIONEER data trust committee, a lay group made up of volunteer members of the public, who supported the use of their data for this study.

### Missing data

Where the proportion of missing data for a given variable is <10%, cases with missing data will simply be excluded from the analysis. Where the degree of missing data is higher, the randomness of the missing data will be assessed, and, if appropriate, multiple imputation is performed. If the degree of missing data for any variable was greater than 20%, the variable will not be included in the analysis.

### Statistical analysis

Data analysis will be performed using the ‘R’ software package (version 4.3) ^13^. The code used will be saved and published along with the resulting publications.

### Statistical analysis plan

Prior to conducting the analysis below, cases meeting each of the coding criteria, clinical definition, or both, will be identified within a smaller cohort of patients from one of the included hospitals. Of these, manual chart review of these patients by respiratory physicians will determine what proportion were treated clinically as HAP, or meet international definitions of HAP ^5^

***Aim 1 – Characterising the demographic and clinical characteristics of patients treated for HAP***

Patient demographics and baseline data will be graphically visualized and characterized using descriptive statistics. For continuous data, mean and standard deviations will be calculated where data are normally distributed and median and interquartile range where not. Categorical data are presented as proportions/frequencies. The included variables are age, sex, ethnicity, place of residence before admission, comorbidities (including the Charleson comorbidity index), medications on admission, and reasons for admission. In addition to reviewing the proportion of cases associated with specific reasons for admission, these cases will be split into elective and emergency admissions.

Where possible, among patients meeting the clinical definition, the date and time of diagnosis will be approximated based on the time of antibiotic prescription. For these patients, the associated vital signs (heart rate, blood pressure, respiratory rate, oxygen saturation, temperature, and mental status) and blood test results (hemoglobin, hematocrit, platelet count, white cell count and differential, CRP, sodium, urea, albumin, and liver enzymes) within 24 h will also be characterized.

The outcome measures are all-cause readmission rates within 1 year of discharge, inpatient mortality, and 30-day and 1-year mortality rates. PIONEER data captures deaths both in and out of hospital, based on primary care records, allowing this to be accurately measured. As secondary outcome measures we will also assess the length of stay and rates of Clostridioides difficile infection as a possible adverse consequence of antibiotic use. Kaplan-Meier survival curves will also drawn, and log-rank tests performed to compare differences between groups (i.e., between non-ventilated HAP and ventilator-associated pneumonia, and between empirical vs. organism-guided therapy).

***Aim 2 – Characterise the microbiology of HAP, to include the rates of microbiological sampling, organisms identified, and whether these were covered by antimicrobials given***

Descriptive statistics will again be used to characterize microbiological sampling rates, focusing on sputum, endotracheal aspirates, bronchoalveolar lavage samples, pleural fluid, blood culture and nasopharyngeal swabs. This will include the type of sample used and tests performed (for example, culture or PCR). Antimicrobials given will be listed individually, then grouped by spectrum of activity to include gram-positive, gram negative, anti-MRSA and anti-pseudomonal cover.

The proportion of patients in whom a potential pathogen is identified – namely a micro-organism known to potentially cause pneumonia, reported in morcrobiology results to a species or genus level - will also be calculated, and the identified pathogens will be compared to the antibiotics that patients received, including dose prescribed. It is acknowledged that organisms identified may relate to colonisation rather than infection however detailed analysis to ascribe likely causation on a patient by patient basis will not be feasible.

The proportion of patients for whom antibiotics were changed (whether de-escalated, stopped, or targeted) after microbiological results were available will be calculated. This will be used to simulate the number of patients who could potentially have antibiotics altered in different scenarios based on increased organism identification rates. We will also compare outcomes for patients depending on whether or not there was resistance to initial antibiotics prescribed.

***Aim 3 - Compare changes in the above between patients with NV-HAP and VAP, and over time***

Trends in the clinical and microbiological variables over time will be compared based on the year of admission. This is of particular relevance, given the inclusion of the covid-19 pandemic within the selected timeframe. A comparison will also be performed between VAP and NV-HAP. The chi-squared test will be used for categorical data, and the t-test or Mann-Whitney test will be used for continuous data. Log-rank tests will be performed to compare differences in survival between VAP and NV-HAP based on Kaplan-Meier curves.

Where the time of diagnosis can be approximated, VAP will be defined as any case where the onset of pneumonia occurs >48hours after ventilation. Where this is not possible (for example, where patients are included based on coding data alone), they will be labelled as VAP if they were ventilated at any point during admission.

***Aim 4 - Determine factors associated with the likelihood of sending samples for microbiological analysis, and with organisms being positively identified***

Multiple logistic regression analysis will be performed to identify factors associated with both sending microbiological samples for analysis and obtaining positive microbiology results among those sent.

A stepwise regression approach will be used to identify the most important predictors, with co-variates considered including those identified in aim 1, together with the setting in which pneumonia occurred (surgical ward, medical ward, or intensive care unit) and time of diagnosis (split between ‘in hours’ (between the hours of 8 and 5)’ and ‘out of hours’. It is hypothesized that the factors affecting the sending of samples include both patient and healthcare setting factors. Patient factors may include the ability of patients to produce samples and the severity of illness, while system factors may include prioritization, staffing levels, and experience within a unit, as well as access to procedures such as bronchoscopy to obtain specimens. These co-variates, while unlikely to be comprehensive, have been selected as likely related to the ability to produce samples, the severity of infection, or system-related variability. The relative impact of each may vary between NVHAP and VAP.

***Aim 5 - Identify factors influencing prognosis in HAP, including comparing outcomes in patients treated with organism-guided antibiotics vs those in whom empirical antibiotics alone were used***

The outcome measures used to assess prognostic factors are mortality within 3 and 12 months and readmission rates within 12 months. All cause readmissions have been chosen rather than those related to infection only, as the potential sequelae of HAP include a generalized increase in frailty that may lead to admission for many different reasons.

In each case, a Cox proportional hazard regression model will be used to investigate the prognostic factors. The factors used as covariates are those identified in aim A, as well as the presence or absence of a microbiological diagnosis as a further categorical variable.

The predictive performance of the existing severity scores will be assessed using the area under the receiver operating characteristic curves. The scores to be assessed included pneumonia-specific scores (CURB-65 and PSI) and general severity scores (NEWS2 and SOFA). Waterlow score is a clinical tool used to predict the development of pressure ulcers and is routinely recorded for all inpatients. However, many of its components (such as age, mobility status, and neurological deficit) are likely to impact the prognosis of patients with HAP; therefore, the Waterlow score will also be investigated for its prognostic value.

In particular, we are focused on the relative impact of obtaining a microbiological diagnosis on the outcome. However, this is influenced by several confounding variables. As a further analysis of the potential impact of this on prognosis, propensity scores will be calculated, and inverse probability of treatment weighting used to create a pseudo-population in which confounding variables are distributed equally between the control and treatment arms. Covariate balance between the groups will be assessed after adjustment using standardized mean differences. The Co-variates used for propensity scoring will be those used in Aim 4. Differences in outcome measures will then be compared between the groups using a Cox proportional hazards analysis.

***Aim 6 - Assess - where it can be established through natural language processing of radiological reports - the difference in outcomes among patients with consolidation and those without***

Natural language processing of radiology reports will be conducted to identify patients where consolidation is present, based on radiologist written reports. Methods used for natural language processing, and steps taken to validate results, will be presented together with results. If natural language processing of radiology reports can identify patients in whom consolidation was present, a sensitivity analysis of the impact of this on mortality and readmission rates will be performed.

## Discussion

This protocol provides the basis for insight into an important and understudied disease. Access to the Health Data Research UK PIONEER database provides a large volume of data, with preliminary searches indicating a population of several thousand patients treated for HAP within the 5 year-frame specified. This is combined with high granularity of data, allowing detailed analyses to be conducted. However, there are several limitations inherent in the study design that may lead to bias in the results.

First, and common to much research on HAP, there is uncertainty that all included patients do indeed have HAP. This arises because of the difficulty in making a clinical diagnosis of HAP as well as limitations within coding data. It is well known that coding data for HAP has high rates of both false positives and false negatives ^6, 14, 15^. The included clinical definition aims to mitigate this by identifying additional cases, and a similar surveillance definition was previously found to have incidence and mortality comparable to estimates of HAP from manual case reviews ^11^. However, this clinical definition still does not help to exclude false positives, and it is acknowledged that there will be significant heterogeneity within the identified cohort. This is not an insignificant limitation. However, this also reflects data available in practice.

Second, coding data will not provide the date or time of diagnosis. An episode of HAP can occur at any point during hospital admission, and patients’ risk factors, medications, and clinical status will vary significantly during that time. Without the date and time of diagnosis, it is not possible to incorporate many variables that might have an impact on outcomes. Therefore, for these it will be necessary to include only patients meeting the clinical surveillance criteria, where the time of diagnosis can be estimated based on the recorded times of prescriptions. This reduces the power of the analysis and may reflect a biased subset of HAP patients if those meeting the surveillance criteria are not representative of the entire HAP population.

Third, while the PIONEER data hub provides access to a large volume of data from a diverse group of patients, all patients are treated within one large NHS trust encompassing four urban hospitals in the UK. This may limit the applicability of findings to sites in other settings or countries, where clinical practice, antimicrobial guidelines, organisational culture, and access to investigations may vary.

## Ethics and consent

This study will use unconsented, anonymous health data, with the need for individual participant consent waived by the ethical approval committee. All study activity is approved by the East Midlands–Derby REC (reference: 20/EM/0158). Specific approvals have been provided by East Midlands–Derby REC (reference: 20/EM/0158) to use unconsented, anonymized health data. The project was also reviewed and approved by the PIONEER Data Trust Committee. Ethical approval was gained on 28/06/2024. 

## Dissemination

The results will be submitted for publication in peer-reviewed journals and for presentations at national and international conferences.

## Data Availability

Following study completion, data access will be provided by PIONEER and can be accessed through a data access request by contacting pioneer@uhb.nhs.uk. The list of antibiotics used to identify patients meeting clinical criteria has been deposited within Figshare, https://doi.org/10.6084/m9.figshare.30102796.v1 ^16^ Data are available under the terms of the Creative Commons 1.0 Universal License (CC0)
