# Supplementary material for: Study Protocol: A retrospective observational analysis of patients treated for hospital-acquired pneumonia
Source: NIHR Open Res. 2025 Sep 15;5:36. Originally published 2025 Apr 23. [Version 2] doi: 10.3310/nihropenres.13853.2 (PMC12464530; doi:10.3310/nihropenres.13853.2)
Supplement: Peer review history

# Reviewer report: Reviewer response for version 2

**DOI:** 10.3310/nihropenres.15343.r37148

**Published:** 2025-09-25

**Authors:** Milan Kolar

**Affiliations:** 1 Palacký University Olomouc, Olomouc, Czech Republic

First of all, I would like to thank the authors of the manuscript for revising the text based on my comments.

 The manuscript has been adequately edited, and I am now pleased to recommend it for acceptance.

Is the study design appropriate for the research question?

Yes

Is the rationale for, and objectives of, the study clearly described?

Yes

Are sufficient details of the methods provided to allow replication by others?

Partly

Are the datasets clearly presented in a useable and accessible format?

Partly

Reviewer Expertise:

Clinical microbiology, AMR, antibiotics, antibiotic therapy

I confirm that I have read this submission and believe that I have an appropriate level of expertise to confirm that it is of an acceptable scientific standard.

# Reviewer report: Reviewer response for version 2

**DOI:** 10.3310/nihropenres.15343.r37149

**Published:** 2025-09-25

**Authors:** Anthony D Bai

**Affiliations:** 1 Queen's University, Kingston, Ontario, Canada

The authors have done a great job addressing my comments and concerns. I do not have any additional comments or suggestions.

Is the study design appropriate for the research question?

Yes

Is the rationale for, and objectives of, the study clearly described?

Yes

Are sufficient details of the methods provided to allow replication by others?

Partly

Are the datasets clearly presented in a useable and accessible format?

Not applicable

Reviewer Expertise:

Infectious diseases, clinical research, analysis of hospital databases

I confirm that I have read this submission and believe that I have an appropriate level of expertise to confirm that it is of an acceptable scientific standard.

# Reviewer report: Reviewer response for version 1

**DOI:** 10.3310/nihropenres.15054.r36716

**Published:** 2025-09-04

**Authors:** Milan Kolar

**Affiliations:** 1 Palacký University Olomouc, Olomouc, Czech Republic

This retrospective study analyzes patients treated for hospital-acquired pneumonia (HAP), distinguishing between ventilator-associated (VAP) and non-ventilator-associated (NV-HAP) forms. The topic is highly relevant and clinically significant, particularly in light of the increasing prevalence of antimicrobial resistance (AMR) and the need to optimize diagnostic and therapeutic strategies. Importantly, inadequately treated HAP may progress to sepsis and septic shock. I believe the study has the potential to provide valuable insights for clinical practice and further research.

 Strengths of the study:

- The focus on NV-HAP, which remains underexplored, is highly beneficial.
- The use of the extensive PIONEER database enables analysis of a large patient cohort with HAP.
- The study is methodologically well-designed, including appropriate statistical methods and chest-imaging based outcome analysis.

Comments and Recommendations:

 1. Microbiological protocol

- The types of clinical samples to be evaluated are not clearly specified. For VAP, lower respiratory tract specimens (e.g., endotracheal aspirate, BAL) are assumed, but this should be explicitly stated. What types of samples will be analyzed in NV-HAP? How will clinically significant findings be distinguished from colonization? I recommend clarifying the criteria for evaluating microbiological results.
- PCR test results are mentioned in the protocol. The authors should specify which results and from which clinical materials.
- I recommend to consider including positive blood cultures as a potential diagnostic criterion.
- The definition of a “putative pathogen” should be clarified, including criteria for distinguishing contaminants from true bacterial pathogens.

2. Antibiotic treatment and AMR

- The study should include not only the types of antibiotics used but also their dosages, as this can significantly influence treatment outcomes and resistance development.
- The term “antibiotic rationalization” should be clearly defined—does it refer to de-escalation, targeted therapy, or treatment discontinuation?
- I recommend analyzing bacterial resistance and its impact on HAP treatment outcomes. Also, assess the effect of inadequate initial antibiotic therapy (i.e., pathogen resistant to the initial treatment) compared to adequate initial therapy (i.e., pathogen susceptible to the administered antibiotic).

3. Inflammatory markers

- I recommend expanding the protocol to include monitoring of inflammatory markers, particularly procalcitonin (PCT), which may aid in decisions regarding initiation and discontinuation of antibiotic therapy.
- Evaluating the dynamics of CRP, leukocytosis, and other laboratory parameters could contribute to better patient stratification.

4. NV-HAP vs. VAP

- The study appropriately distinguishes between these two groups but should further reflect differences in diagnostic method availability and sampling feasibility.
- Consider evaluating access to specialized procedures (e.g., bronchoscopy) as a systemic factor influencing diagnostic accuracy.

Conclusion:

 The study represents a significant contribution to the understanding of HAP, particularly NV-HAP. To enhance its clinical relevance, I recommend incorporating the above points into the study protocol.

Is the study design appropriate for the research question?

Yes

Is the rationale for, and objectives of, the study clearly described?

Yes

Are sufficient details of the methods provided to allow replication by others?

Partly

Are the datasets clearly presented in a useable and accessible format?

Partly

Reviewer Expertise:

Clinical microbiology, AMR, bacterial infections, antibiotic therapy

I confirm that I have read this submission and believe that I have an appropriate level of expertise to confirm that it is of an acceptable scientific standard, however I have significant reservations, as outlined above.

# Response

**Published:** 2025-09-09

**Authors:** Samuel Quarton

**Affiliations:** NIHR Birmingham Biomedical Research Centre, Birmingham, England, UK

Many thanks for your thorough and helpful appraisal of our proposed study. It is encouraging to hear you feel the study will be beneficial, and we value that you have highlighted some of the strengths of the proposed study.

 Please see below for our responses to your specific comments, together with the changes made in light of these. The updated version of the text has been submitted and will be visible for review shortly. 

 1. Microbiological protocol

- The types of clinical samples to be evaluated are not clearly specified. For VAP, lower respiratory tract specimens (e.g., endotracheal aspirate, BAL) are assumed, but this should be explicitly stated. What types of samples will be analyzed in NV-HAP? How will clinically significant findings be distinguished from colonization? I recommend clarifying the criteria for evaluating microbiological results.

Thank you for highlighting this. We have altered this within the updated version of the text to specify “sputum, bronchoalveolar lavage samples, pleural fluid, blood culture and nasopharyngeal swabs”.

 Distinguishing findings from colonisation is a challenge across pneumonia diagnosis generally and would not be possible to accurately perform without a case-by-case analysis of other clinical and radiological results for each patient, which would not be feasible across the dataset. For the purposes of this study we will count any bacteria identified, reviewed by microbiology and reported to clinicians to a genus or species level as a potential pathogen. (Where there is mixed growth or upper-respiratory tract organisms identified only, these are reported as such in our hospital, rather than to a species level. The text has been updated to reinforce these are potential pathogens only, and may represent colonisation.

  

- PCR test results are mentioned in the protocol. The authors should specify which results and from which clinical materials.

Samples are those stated above. Any microbiological PCR results performed on these samples will be collected, including for specific organisms or as part of multiplex panels.

  

- I recommend to consider including positive blood cultures as a potential diagnostic criterion.

We will be examining rates of blood culture positivity.

  

- The definition of a “putative pathogen” should be clarified, including criteria for distinguishing contaminants from true bacterial pathogens.

As above – we have expanded on this in the text, mainly to explain the limitation, as in practice distinguishing between true pathogens from contaminants/colonisation will not be feasible.

  

- The study should include not only the types of antibiotics used but also their dosages, as this can significantly influence treatment outcomes and resistance development.

This is a helpful suggestion and has been added to the text.

  

- The term “antibiotic rationalization” should be clearly defined—does it refer to de-escalation, targeted therapy, or treatment discontinuation?

Thank you for this – we aim to capture any change that has been guided by organism identification, to include de-escalation, stopping treatment, and escalating/targeting treatment to better cover identified pathogens. We have edited the text to reflect this.  

  

- I recommend analyzing bacterial resistance and its impact on HAP treatment outcomes. Also, assess the effect of inadequate initial antibiotic therapy (i.e., pathogen resistant to the initial treatment) compared to adequate initial therapy (i.e., pathogen susceptible to the administered antibiotic).

This is a very important suggestion. We had intended to conduct this analysis but realise it is not explicitly stated within the text. Have now added this.

  

- I recommend expanding the protocol to include monitoring of inflammatory markers, particularly procalcitonin (PCT), which may aid in decisions regarding initiation and discontinuation of antibiotic therapy.

We are including white cell count and CRP as markers of inflammation. While PCT would be helpful, in reality only a very small minority of patients are likely to have this available within our cohort, especially among the ward-based NVHAP group.

  

- Evaluating the dynamics of CRP, leukocytosis, and other laboratory parameters could contribute to better patient stratification.

Agreed, this will be interesting to review. We have included these laboratory parameters among the variables for our analysis.

                               4. NV-HAP vs. VAP

- The study appropriately distinguishes between these two groups but should further reflect differences in diagnostic method availability and sampling feasibility.
- Consider evaluating access to specialized procedures (e.g., bronchoscopy) as a systemic factor influencing diagnostic accuracy.

Have added statements around availability of bronchoscopy, and the variability in access to diagnostic approaches among NVHAP and VAP.

 Many thanks again for your time and consideration in reviewing our manuscript.

# Reviewer report: Reviewer response for version 1

**DOI:** 10.3310/nihropenres.15054.r35522

**Published:** 2025-05-27

**Authors:** Anthony D Bai

**Affiliations:** 1 Queen's University, Kingston, Ontario, Canada

This is a study protocol for a retrospective cohort study on hospital acquired pneumonia using hospital electronic database. The objectives are to compare ventilated and non-ventilated cases in terms of microbiologic diagnosis and how microbiologic diagnosis impact mortality. The study uses the data from 4 hospitals within the University Hospital Birmingham NHS Trust. 

 Overall, I think it is a worthwhile study given that non-ventilated HAP is not well characterized or studied. It would also be worthwhile to understand the practices such as microbiologic work-up for these patients and see if there are any association between practice patterns and patient outcomes with the hope that it may lead to improvements in quality of care and patient outcome. 

 My main criticism is how the HAP patients are defined. Based on the described methodology, there were 2 ways of how HAP was diagnosed based on diagnosis codes and based on surveillance definition. There would be two different cohorts based on these two different definitions. It is unclear if the subsequent analysis is done on either or both of these cohorts. Either way will have a significant number of false positives and negatives. It should be noted that the criteria based on reference 11 has only a kappa of 0.33 in terms of agreement with clinician diagnosis. I think it may be worthwhile to try to do manual chart review on a proportion of cases and have clinicians determine which patients had HAP. Then see how this compares to the above 2 ways to define VAP. This would provide some important internal validation and can help decide which way to define the cohort between doing the rest of the analysis. 

 Major comments: 

 - Protocol, Data sources and variables: For the criteria based on reference 11, criteria on new antibiotic prescription, the antibiotic should be specified. The original criteria used a list of antibiotics (in their eTable 1) that are used empirically for HAP. This should be specified in this protocol and this antibiotic list may change depending on the England guidelines of what is appropriate antibiotic therapy for HAP

 - Statistical analysis plan Aim 1: Can the authors justify the rationale for all-cause readmission within 1 year of discharge? This seems like a very long follow-up for readmission. I am not sure how a readmission at end of 1 year post discharge would be related to the original HAP. Note that within 1 year of discharge would extend follow-up to beyond 1 year given discharge would occur after HAP diagnosis. 

 - Statistical analysis plan Aim 1: For 30-day and 1-year mortality, would the database be able to capture out-of-hospital deaths? If so, it should be specified how this can be captured and how accurate this would be. 

 - Statistical analysis plan Aim 1: Nosocomial infections potentially related to antibiotic such as Clostridium difficile - this should be defined better. 

 - Statistical analysis plan Aim 2: The microbiological sampling considered in the study should be specified. Sputum and bronchoscopy samples are a given, but what about nasopharyngeal swab or blood culture?

 - Statistical analysis plan Aim 2: How will putative pathogen be defined? It will need interpretation of the culture results, as culture may yield multiple organisms and many organisms can be considered contaminants (e.g. candida, oral flora bacteria). 

 - Statistical analysis plan Aim 2: How will the study describe antimicrobial used? Would the antimicrobial agents be listed individually or in groups? I would suggest to describe group of antibiotics based on coverage such as anti-MRSA coverage, anti-pseudomonal coverage etc. 

 - Statistical analysis plan Aim 2: I don't understand what rationalized mean in "The proportion of patients for whom antibiotics were rationalized following microbiological sampling will be calculated." Please explain. 

 - Statistical analysis plan Aim 2: Sometimes a culture may be helpful even if it does not grow anything. For example, in a patient whose sputum gram stain showed no gram-positive cocci and culture did not grow MRSA, then some clinicians may discontinue MRSA coverage. The authors should consider accounting for this and look at if culture taken (whether positive or negative) can lead to narrowing of antimicrobials. 

 - Statistical analysis plan Aim 4: One important system factor would be if the hospitals have respirology / pulmonology service and bronchoscopy availability

 - Statistical analysis plan Aim 5: I understand the rationale for this objective. However, one criticism to anticipate is that there is clearly confounding by indication with respect of obtaining cultures on a patient. There is a reason that clinicians ordered sputum cultures or performed bronchoscopy on one patient but not in the other. In the end, these two groups will be fundamentally different that the propensity score weighting will not be able to balance them and there will always be residual confounding. 

 - Statistical analysis plan Aim 5: There should be description of how the outcomes will be compared between the two groups after propensity score weighting. 

 - Statistical analysis plan Aim 6: There should be description of how natural language processing will be done and how the gold standard of consolidation is done. 

 Minor comments

 - The study period from 2018 to 2022 is a bit outdated even considering the 1-year follow-up, can the study period be extended up to 2023 or 2024? 

 - Note that C difficile should be **Clostridioides** difficile

 Minor edits

 - Introduction: "Although the significance of HAP is widely **z** recognised" - typo

 - Introduction: " **y** these have been found to be absent in up to half of patients treated with HAP" - typo

 - Introduction: "pneumonia occurring among non-ventilated **z** hospitalised patients (NV-HAP)." - typo

 - Introduction: "This may not be **possiblebecause** many of the factors predisposing patients" - needs space between possible and because

 - Aims: "Characterise the microbiology of HAP, to include the rates of microbiological sampling, the organisms identified, and whether these were covered by **antimicrobialsgiven** " - needs space between antimicrobials and given

 - Patient and Public Involvement: "their perspective on what to **zprioritise** " - typo

 - Discussion:  "clinical status **willvary ** significantly during that time." - needs space between will and vary

 - Discussion: "Therefore, **these ** it will be necessary" - delete these

Is the study design appropriate for the research question?

Yes

Is the rationale for, and objectives of, the study clearly described?

Yes

Are sufficient details of the methods provided to allow replication by others?

Partly

Are the datasets clearly presented in a useable and accessible format?

Not applicable

Reviewer Expertise:

Infectious diseases, clinical research, analysis of hospital databases

I confirm that I have read this submission and believe that I have an appropriate level of expertise to confirm that it is of an acceptable scientific standard, however I have significant reservations, as outlined above.

# Response

**Published:** 2025-09-09

**Authors:** Samuel Quarton

**Affiliations:** NIHR Birmingham Biomedical Research Centre, Birmingham, England, UK

Many thanks for your thorough and helpful appraisal of our proposed study. It is encouraging to hear you feel the study is of value, and your comments and input are greatly appreciated. 

 Please see below for our response to your specific comments, together with changes based on your insights. The updated version has been submitted and will be visible shortly. 

*My main criticism is how the HAP patients are defined. Based on the described methodology, there were 2 ways of how HAP was diagnosed based on diagnosis codes and based on surveillance definition. There would be two different cohorts based on these two different definitions. It is unclear if the subsequent analysis is done on either or both of these cohorts. Either way will have a significant number of false positives and negatives. It should be noted that the criteria based on reference 11 has only a kappa of 0.33 in terms of agreement with clinician diagnosis. I think it may be worthwhile to try to do manual chart review on a proportion of cases and have clinicians determine which patients had HAP. Then see how this compares to the above 2 ways to define VAP. This would provide some important internal validation and can help decide which way to define the cohort between doing the rest of the analysis.*

**Response: **We agree that this is an important limitation. At present we are keen to include both cohorts – diagnostic coding as this is the primary way in which cases are retrospectively identified, and the surveillance definition due to known limitations in the coding data, and due to an ability to associate this with a date and time for analysis of time-dependent variables.  

 Sensitivity analysis will be performed - comparing patients who meet the coding definition, those meeting the surveillance definition, and those meeting both. We have added a statement regarding this in the manuscript.

 Internal validation would be helpful, however given limitations in the anonymised available data, accessing patient records to compare with a manual review is not possible.

 However, we will separately use both definitions on a smaller dataset from one of the four hospital sites where case review is possible and perform manual case review among this group. While this will not be as helpful as random sampling from within the cohort used for analysis, it will give some indication of the utility of these definitions among our patients.   

*- Protocol, Data sources and variables: For the criteria based on reference 11, criteria on new antibiotic prescription, the antibiotic should be specified. The original criteria used a list of antibiotics (in their eTable 1) that are used empirically for HAP. This should be specified in this protocol and this antibiotic list may change depending on the England guidelines of what is appropriate antibiotic therapy for HAP*

**Response: **We agree and apologies for this omission. The list of included antibiotics is now included. We have based our list on antibiotics used empirically for HAP, with reference to the included citation, NICE guideline 139 (Pneumonia (hospital-acquired): antimicrobial prescribing), and local trust guidelines.

 As a further screen, where patients met the criteria based on antibiotics that did not meet local guidance, but with potential use as empirical treatment for HAP, and where these affected >100 possible cases, review of case notes will be performed to identify in what proportion HAP is mentioned. Where fewer than 50% of cases reference HAP, these antibiotics will not be included in the final list.

*- Statistical analysis plan Aim 1: Can the authors justify the rationale for all-cause readmission within 1 year of discharge? This seems like a very long follow-up for readmission. I am not sure how a readmission at end of 1 year post discharge would be related to the original HAP. Note that within 1 year of discharge would extend follow-up to beyond 1 year given discharge would occur after HAP diagnosis.*

**Response: **While 1 year follow up is a relatively long period of time, we believe it is justified. Pneumonia has previously been shown to have lasting impacts on outcome ( Long-term prognosis in community-acquired pneumonia - PMC). The cohort at greatest risk of HAP is older and more co-morbid, both associated with frailty. HAP may plausibly lead to a step-change in increased frailty, and this may be captured in following up all-cause readmissions for up to 1 year.

*- Statistical analysis plan Aim 1: For 30-day and 1-year mortality, would the database be able to capture out-of-hospital deaths? If so, it should be specified how this can be captured and how accurate this would be.*

**Response: **Added wording to clarify this – yes we capture out-of-hospital deaths using primary care data linked to official registers of deaths.

*- Statistical analysis plan Aim 1: Nosocomial infections potentially related to antibiotic such as Clostridium difficile - this should be defined better.*

**Response: **Thank you for the feedback. This has been reworded.

*- Statistical analysis plan Aim 2: The microbiological sampling considered in the study should be specified. Sputum and bronchoscopy samples are a given, but what about nasopharyngeal swab or blood culture?*

**Response: **Updated to specify this. Sputum, bronchoscopy samples, pleural fluid, blood culture and nasopharyngeal swabs will all be captured and analysed, together and separately.

*- Statistical analysis plan Aim 2: How will putative pathogen be defined? It will need interpretation of the culture results, as culture may yield multiple organisms and many organisms can be considered contaminants (e.g. candida, oral flora bacteria).*

**Response: **Within our hospital sites oral flora bacteria are reported in those terms – ie “mixed oral flora” or “mixed upper respiratory tract flora only”, rather than as specific organisms identified. There remains an inability to differentiate between colonisation/contamination and infection from microbiology report alone, however more detailed analysis on a patient-by-patient basis would not be feasible across the whole dataset. For the purposes of this study we will count any bacteria identified, reviewed by microbiology and reported to clinicians to a genus or species level as a potential pathogen. Rates of candida or other environmental fungi identified will be reported but not used as part of this analysis, which is focused primarily on the spectrum of antibiotic usage. Text changed to clarify this, and reinforce these are potential pathogens only.

*- Statistical analysis plan Aim 2: How will the study describe antimicrobial used? Would the antimicrobial agents be listed individually or in groups? I would suggest to describe group of antibiotics based on coverage such as anti-MRSA coverage, anti-pseudomonal coverage etc.*

**Response: **We will group antibiotics by spectrum of activity, and compare proportions received of each group in patients with and without an organism identified. Text updated to clarify this.

*- Statistical analysis plan Aim 2: I don't understand what rationalized mean in "The proportion of patients for whom antibiotics were rationalized following microbiological sampling will be calculated." Please explain*

**Response: **We agree this could be worded more clearly. We aim to capture what proportion of patients had their antimicrobial regime changed in light of microbiological results, for example to a narrower-spectrum antibiotic, or changed to add additional cover. Text updated to hopefully make this more explicit and specific.

*- Statistical analysis plan Aim 2: Sometimes a culture may be helpful even if it does not grow anything. For example, in a patient whose sputum gram stain showed no gram-positive cocci and culture did not grow MRSA, then some clinicians may discontinue MRSA coverage. The authors should consider accounting for this and look at if culture taken (whether positive or negative) can lead to narrowing of antimicrobials.*

**Response: **Thank you for this suggestion. In addition to the impact of positive microbiology, we will indeed examine the impact on antimicrobial prescribing of taking cultures. Text updated to reflect this.

*- Statistical analysis plan Aim 4: One important system factor would be if the hospitals have respirology / pulmonology service and bronchoscopy availability*

**Response: **This is a key system factor, however all hospitals included in the analysis have these services available on site.

*- Statistical analysis plan Aim 5: I understand the rationale for this objective. However, one criticism to anticipate is that there is clearly confounding by indication with respect of obtaining cultures on a patient. There is a reason that clinicians ordered sputum cultures or performed bronchoscopy on one patient but not in the other. In the end, these two groups will be fundamentally different that the propensity score weighting will not be able to balance them and there will always be residual confounding.*

**Response: **We understand – and largely agree with – this criticism. However, clinician request is likely not the only factor impacting whether cultures are sent, something which will hopefully be illuminated by aim 4.

 Further, as the ATS 2016 guidelines recommend all patients with suspected HAP undergo culture of lower respiratory tract secretions, we believe the indication for sputum culture should be present for all included patients. Sputum and bronchoscopy samples will be assessed separately, as we agree that clearly patients who are selected to undergo bronchoscopy will represent a very different cohort from those who are not. Among the NV-HAP cohort, very few are anticipated to have undergone bronchoscopy. Despite the clear challenges with confounding, we believe this is an important area to examine.

*- Statistical analysis plan Aim 5: There should be description of how the outcomes will be compared between the two groups after propensity score weighting.*

**Response: **Mortality will be assessed with a Cox proportional hazards analysis. Added to the text to clarify.

*- Statistical analysis plan Aim 6: There should be description of how natural language processing will be done and how the gold standard of consolidation is done.*

**Response: **Methodology for this is under development. We have added that the gold standard we are using is radiologist reports, and that we will provide information on methods to develop and validate results with any subsequent results paper.

*Minor edits*

**Response: **Thank you for flagging these. Several of these typos, plus additional inconsistencies in tense, seem to have been introduced during the copyedit process, for which we apologies.
